# Toxic shock syndrome responsive to steroids

**DOI:** 10.1186/1752-1947-1-5

**Published:** 2007-02-16

**Authors:** Nikhil Vergis, David A Gorard

**Affiliations:** 1 Wycombe Hospital, Queen Alexandra Road, High Wycombe, Bucks HP11 2TT, UK

## Abstract

**Background:**

Toxic Shock Syndrome is a dangerous disease with clinical features mimicking bacterial sepsis. The best management of Toxic Shock Syndrome is not determined.

**Case presentation:**

A 28 year-old woman presenting with high fever, tachycardia and widespread erythroderma is described. She failed to respond to intravenous antibiotics and required ITU admission. High dose corticosteroids dramatically improved her clinical condition.

**Conclusion:**

Toxic Shock Syndrome should be considered in the differential diagnosis of unexplained fever, rash and features resembling septic shock. Corticosteroids should be considered in the treatment of Toxic Shock Syndrome.

## Background

Toxic Shock Syndrome (TSS) is a super-antigen mediated, potentially fatal disease [1]. Its rarity ensures it is often considered late in the clinical course of the disease, and controlled trials on the best management are lacking. A case of TSS is described; the illness was refractory to intravenous antibiotics but successfully treated with intravenous steroids.

## Case report

A 28-year-old woman presented to A&E with a short history of diarrhoea and vomiting associated with high fevers, sore throat and flushing of her skin. There had been no recent foreign travel, exposure to toxins or drugs, nor gynaecological symptoms. There had been no recent use of tampons. She had not been menstruating while using 3-monthly intramuscular injections of Depo-Provera, a long-acting progesterone, as contraception.

Interestingly, 6 years earlier she had been admitted with a toxic shock-like syndrome to another hospital and required ITU care. At that time and without serological confirmation, it was presumed that a staphylococcal or possibly streptococcal infection had triggered her condition.

On examination she looked unwell. She was distressed and flushed with widespread erythema of her skin. Her temperature was raised at 39.6°C and pulse elevated at 120 beats per minute with regular rhythm. Her blood pressure was maintained at 110/70 mmHg. Cardiac auscultation was normal, as was the rest of the clinical examination. A subsequent pelvic examination was normal.

Blood tests showed a white cell count of 9.4 × 10^9^/l with left shift of neutrophils. The CRP and ESR were raised at 250 ng/ml and 45 mm/hr respectively. Arterial blood gases demonstrated respiratory alkalosis with *p*H 7.57, *p*O_2 _13.8 kPa and *p*CO_2 _2.2 kPa. A chest radiograph was normal.

She received aggressive intravenous fluid resuscitation. After blood was drawn for culture, intravenous cefuroxime and clarithromycin were empirically prescribed for a presumed bacterial septic illness. However the antibiotics failed to control either her pyrexia or her tachycardia. Her rash initially resembled severe sunburn but went on to exfoliate and then desquamate after two days. Dermatology opinion agreed that the skin condition was consistent with a diagnosis of TSS, and supportive treatment recommended. Her serum albumin dropped to 20 g/l during the first few days of her admission. Her temperature remained elevated at 38–39°C. Repeated blood cultures were sterile while stool cultures were negative for bacterial pathogens. All throat, skin and high vaginal swabs yielded no growth. Paired acute and convalescent antistreptolysin 0 titres and antistaphylolysin 0 titres showed no rise. Rheumatoid factor, antinuclear and other autoantibodies were negative. C_1 _esterase inhibitor and complement levels were normal. Screens for viruses, toxic metals, cardiolipin antibody, urinary porphyrins and porphobilinogen were all negative.

Five days after admission her fever rose to 40°C and she became more unwell with delirium. Her pulse rose to140 beats per minute. An echocardiogram was normal. She was transferred to the ITU for further observation. Since no bacteria had been cultured after 5 days and since she had made no response to antibiotics and remained very unwell, a decision was made to empirically administer corticosteroids. This decision was based on anecdotal reports, and a retrospective analysis had suggested possible benefit from corticosteroid use [2]. She was given intravenous methylprednisolone 1 g daily for three days. Administration of this corticosteroid rapidly and dramatically improved her clinical condition with resolution of her temperature and tachycardia. She was converted to oral steroids and discharged home with prednisolone 30 mg daily. The dose was subsequently tapered over 6 weeks at outpatient follow up, and she remains well 2 years later.

## Discussion

TSS is an acute, toxin-mediated febrile illness that can rapidly lead to multisystem organ failure. Its characteristic features of high fever, macular erythrodermic rash (likened to sunburn), myalgia, diarrhoea and other systemic upset were evident in the case reported here. A plethora of toxic proteins have been implicated in its pathogenesis, most notably the Toxic Shock Syndrome Toxin-1 (TSST-1) [1]. This protein, secreted by *S. aureus*, has the ability to cause a remarkable expansion of T lymphocytes displaying specific β chain variable regions of the T-cell antigen receptor: it is this property that earns TSST-1 classification as a superantigen. Superantigens bypass normal antigen presentation and stimulate over 20% of the body's T-cells, inducing the massive release of various cytokines, prostaglandins and leukotrienes and initiating a dangerous inflammatory response.

The first reports of TSS emerged in 1978 [3]. A statistical association between tampon use and the development of TSS in women, the recognition that asymptomatic vaginal ulceration occurs in tampon users, and that *S aureus *colonises the normal vaginal flora of 5% of women, led to the hypothesis that ulceration of the vaginal mucosa resulting from tampon use may provide a common point of entry for the *S aureus *exotoxin. Since these toxins can enter the bloodstream from various different portals, not just the vaginal mucosa, TSS-like presentations have subsequently been described in women who are not menstruating, and in men.

TSS can therefore be divided into menstrual and nonmenstrual subgroups, with around 45% of all cases being nonmenstrual in origin. Three basic features are thought to be required to develop TSS: i) patient colonisation or infection with *S aureus*, ii) production of TSST-1 or similar toxins by the bacterium, and iii) an entry route for the toxins into the circulatory system. It should be noted that *S aureus *bacteraemia has been found to have no focus in up to one third of cases [4]. Definite TSS requires the presence of fever, rash, hypotension, multisystem disease, and desquamation, with the latter occurring 1–2 weeks after the onset of illness; absence of 1 criterion constitutes "probable" TSS. Currently, there is no diagnostic test for TSS. Although it is hypothesised that the disease can only manifest in those who are unable to generate sufficient antibody titres to TSST-1, the absence of TSST-1 antibodies does not help in the diagnosis.

In the case we report, convalescent serum samples for streptococcal and staphylococcal antibodies were negative and so the underlying cause of her TSS was not identified. Our patient had experienced TSS six years earlier. While recurrence of menstrual TSS is not unusual, recurrent nonmenstrual TSS as in this report, is rarer [5]. Failure to eradicate *S. aureus *colonization has been the proposed mechanism.

The differential diagnosis of TSS at presentation in A&E is broad. Bacterial infection with associated septicaemia should most rapidly come to mind. The most common of such infections to be considered is acute pyelonephritis; the most serious is meningococcal septicaemia. Both of these should be actively excluded.

Initial management of TSS is supportive, and aggressive fluid resuscitation is essential. High-dose anti-staphyloccocal antibiosis is recommended, and will almost automatically have been given to treat infective conditions in the differential diagnosis. Flucloxacillin is an appropriate antibiotic choice, and it demonstrably inhibits TSST-1 toxin production *in vitro *when combined with gentamicin [6] Pooled human immunoglobulin has also been used in some patients. Corticosteroids have been occasionally used in TSS, and one retrospective series suggested some benefit [2]. Although there are no definitive data to support the use of corticosteroid treatment in TSS, our patient responded well to this treatment. Administration of methylprednisone marked the turning point in her clinical course, presumably by suppressing the inflammatory response associated with TSS. While the super-toxin mediated inflammatory illness in TSS must be clearly distinguished from more frequently seen septic shock illnesses, it is interesting that steroids may controversially have a role in some septic shock patients [7].

In summary, TSS should be included in the differential diagnosis of a patient with a severe toxic illness with associated fever and rash, in the emergency department. Corticosteroids should be considered in the management of TSS.

## Competing interests

The authors declare that they have no competing interests.
